# The Role of Open Access in Reducing Waste in Medical Research

**DOI:** 10.1371/journal.pmed.1001651

**Published:** 2014-05-27

**Authors:** Paul Glasziou

**Affiliations:** Bond University, Robina, Australia

## Abstract

In a guest editorial, Paul Glasziou discusses waste in medical research and how this can be ameliorated through improving post-publication access.

*Please see later in the article for the Editors' Summary*

Twenty years ago an editorial by Doug Altman in the *BMJ* [1], “The Scandal of Poor Medical Research”, decried the poor design and reporting of research, stating that “huge sums of money are spent annually on research that is seriously flawed through the use of inappropriate designs, unrepresentative samples, small samples, incorrect methods of analysis, and faulty interpretation”. Since then, change has been gradual, while the list of problems has lengthened, and documentation of their magnitude has accumulated. Recent years, however, have seen a crescendo of concern. Public awareness has been accelerated with the publication of Ben Goldacre's *Bad Pharma* [2], which clearly articulated the problems posed by biased non-publication and reporting of pharmaceutical research. Wider awareness of these issues helped spark the AllTrials campaign (http://www.alltrials.net/), which asks for “all trials registered; all results reported”. Of course, the problems of poor design and reporting, as well as selective non-publication, extend well beyond drug trials to most areas of research: drug and non-drug, basic and applied, interventional and observational, animal and human. A 2009 paper in *The Lancet* [3] estimated that three problems—flawed design, non-publication, and poor reporting—together meant over 85% of research funds were wasted, implying a global total loss of over US$100 billion per year. This year, a follow-up series [4] more extensively documented this wastage, confirming the earlier estimate, but adding details and a series of more explicit recommendations for action.

The waste sounds bad, but the reality is worse. The estimate that 85% of research is wasted referred only to activities prior to the point of publication. Much waste clearly occurs after publication: from poor access, poor dissemination, and poor uptake of the findings of research. The development of open access to research [5] is important to reduce this post-publication waste. Poor access—including paywalls, restrictions on re-publication and re-use, etc.—limits both researcher-to-researcher and researcher-to-clinician communications. As *PLOS Medicine* editorial leaders pointed out in a PubMed Commons response to the *Lancet* series [6], open access is more than free access and includes “free, immediate access online; unrestricted distribution and re-use rights in perpetuity for humans and technological applications; author(s) retains rights to attribution; papers are immediately deposited in a public online archive, such as PubMed Central” [7]. Globally, the most important access problem is arguably due to language barriers, and with the growth of research in non-English-speaking countries, particularly China, this problem is likely to grow. Language barriers make even free-access research unusable, but by eliminating restrictions on re-publication and re-use, open access can at least reduce barriers to translation.

Solving the problems of pre-publication waste and post-publication access could hugely accelerate medical research. Even the complete solution of these problems, however, would be insufficient to close the research–practice gap. Paradoxically, the plethora of research is itself a barrier to its use. A recent analysis of trials and reviews by specialty found an unmanageable scatter of research [8]. For example, in neurology the annual output was 2,770 trials across 896 journals, and 547 systematic reviews across 292 journals. So, in addition to access, clever systems of synthesis, filtering, findability, and usability are needed if the users of research are to cope with this information deluge [9]. The enormous marketing budgets of pharmaceutical companies demonstrate the importance they place on investing resources in getting the message of their research to decision makers. Unfortunately, little such investment is made in non-commercial research, and this research is consequently neglected. This concern has led to the development of different approaches given names such as “evidence-based medicine”, “knowledge translation”, and “implementation science”.

To get full value from research investment, we need to reduce both the annual US$100 billion of pre-publication (research production) waste and the unquantified cost of post-publication (research dissemination) barriers (Figure 1). Open access will not in itself fix the problems of poor research question selection, poor study design, selective non-publication, or poor or biased reporting, but these can be ameliorated considerably through appropriate editorial policies and peer review processes. Open-access medical journals must maintain particularly high standards for these processes in order to avoid merely increasing access to a biased selection of (often flawed) research. At the same time, improving research quality but keeping access restricted would mean continued waste in the use and uptake of good science.

**Figure 1:**
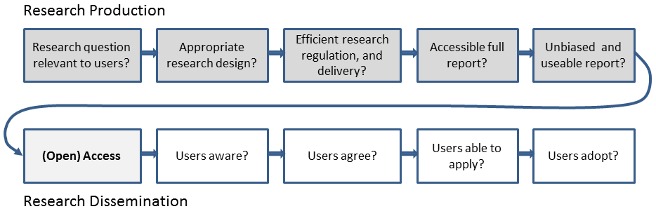
Chain of research production and dissemination.

“As the system encourages poor research,” wrote Altman in 1994 [1], “it is the system that should be changed. We need less research, better research, and research done for the right reasons.” To that must be added a need for research that is communicated effectively to those who need it. If over a 100 billion dollars of medical research money were being wasted by corruption, the public and political outcry would be overwhelming. That resources of this magnitude are being wasted through incompetence and inattention should be seen as a similar scandal. Badly designed and poorly thought through systems of research and dissemination subtract massively from global human health: they demand attention—and action.
